# The influence of lipid digestion on the fate of orally administered drug delivery vehicles

**DOI:** 10.1042/BST20210168

**Published:** 2021-08-25

**Authors:** Ben J. Boyd, Andrew J. Clulow

**Affiliations:** Drug Delivery, Disposition and Dynamics, Monash Institute of Pharmaceutical Sciences, Monash University, 381 Royal Parade, Parkville, VIC 3052, Australia

**Keywords:** digestion, drug delivery, lipid, self-assembly, X-ray scattering

## Abstract

This review will focus on orally administered lipid-based drug delivery vehicles and specifically the influence of lipid digestion on the structure of the carrier lipids and their entrained drug cargoes. Digestion of the formulation lipids, which are typically apolar triglycerides, generates amphiphilic monoglycerides and fatty acids that can self-assemble into a diverse array of liquid crystalline structures. Tracking the dynamic changes in self-assembly of the lipid digestion products during digestion has recently been made possible using synchrotron-based small angle X-ray scattering. The influence of lipid chain length and degree of unsaturation on the resulting lipid structuring will be described in the context of the critical packing parameter theory. The chemical and structural transformation of the formulation lipids can also have a dramatic impact on the physical state of drugs co-administered with the formulation. It is often assumed that the best strategy for drug development is to maximise drug solubility in the undigested formulation lipids and to incorporate additives to maintain drug solubility during digestion. However, it is possible to improve drug absorption using lipid digestion in cases where the solubility of the dosed drug or one of its polymorphic forms is greater in the digested lipids. Three different fates for drugs administered with digestible lipid-based formulations will be discussed: (1) where the drug is more soluble in the undigested formulation lipids; (2) where the drug undergoes a polymorphic transformation during lipid digestion; and (3) where the drug is more soluble in the digested formulation lipids.

## Introduction

### Changes in lipid composition during digestion and associated lipid self-assembly

Nature has developed enzymes as a means of controlling complex structural changes in biomaterials in our body. Lipids provide energy, can be carriers for lipophilic nutrients/drugs and are particularly important enzyme substrates with a wide range of lipases and phospholipases operating to transform lipid species from our diet into absorbable components [1]. The most prominent enzymatic reactions of relevance to oral drug delivery are lipases acting to cleave fatty acid moieties from triglycerides (triacylglycerols/TAGs). The triglycerides consumed in our diet constitute over 95% of our lipid intake. They are critical to the transport of lipophilic nutrients through our blood and lymphatic systems in lipid particles such as lipoproteins and chylomicrons [2]. However, triglycerides are not absorbed intact from our gastrointestinal tract because they have very poor solubility in aqueous environments. As such, they are disassembled by lipases in our gut to form more polar 2-monoglycerides and fatty acids [3]. The absorbed fatty acids and monoglycerides are then re-assembled into triglycerides by intracellular enzymes after absorption for transport through the body. Phospholipases act to disassemble specific parts of phospholipid molecules, most prominently removal of one fatty acid residue to yield lysophospholipids with different surfactant properties [4]. As is the case for triacylglycerol lipases, the cleavage of the fatty acid from the phospholipid enables absorption of the components of phospholipid molecules for re-use by the body. While our understanding of the process of lipid digestion from a biochemical standpoint is therefore well developed, the physical-chemical and structural aspects around the fate of lipids in particular is less well understood.

**Figure 1.**
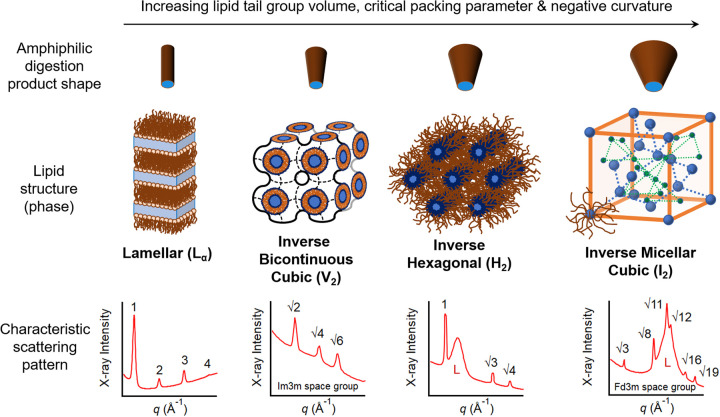
The average shape of the amphiphilic 2-monoglycerides and fatty acids produced during lipid digestion dictates the resulting lipid structures (phases) that form. Each phase has a characteristic X-ray scattering (diffraction) pattern shown beneath their associated phases above. In some of the examples shown, the scattering patterns overlap with the diffraction from calcium soaps formed by binding of calcium in the medium by fatty acids produced during digestion and the broad peaks associated with the calcium soaps are labelled ‘L'. The numbers above each peak indicate the characteristic ratios in peak position that are used to identify the respective phases. Note that there are additional V_2_ and I_2_ packing structures with different space groups and for a more complete list of characteristic scattering patterns the reader is referred to [6]. Adapted from [7] with permission, future requests for reproduction should be made to Elsevier (https://www.sciencedirect.com/science/article/abs/pii/S0268005X20311759).

The production of polar amphiphilic lipids that partition to the lipid-aqueous interfaces of fat droplets during lipid digestion has important consequences for lipid self-assembly in the gut. The critical packing parameter concept provides a roadmap for the likely course of structural transitions that would be expected to occur with increasingly polar lipid compositions [5,6]. The critical packing parameter (CPP) is defined as CPP = V/Al, the ratio of the volume occupied by the hydrophobic tail in lipid domains (V) to the product of the equilibrium headgroup area at the lipid-aqueous interface (A) and the extended length of the hydrophobic tail (l). When this ratio is close to 1, lamellar and vesicular/bilayer type lipid structures form that are locally flat (L_α_, Figure 1). As the volume occupied by the tail group increases relative to the product of the head group area and the tail length, the CPP increases. This leads to a progression of water-in-oil structures from inverse bicontinuous cubic (V_2_, Figure 1) phases comprising crumpled bilayers with interpenetrating water channels, to cylindrical aqueous channels packed into a hexagonal array (H_2_, Figure 1) and finally to inverse micellar (I_2_, Figure 1) phases in which the inverse micelles are packed in a cubic array. The subscript 2 in the identity of these phases highlights that they are inverse or water-in-oil structures, where aqueous channels/cylinders/micelles are ordered within a lipidic continuous phase. These structural changes represent increasing curvature of the lipid-aqueous interfaces towards the aqueous domains with increasing CPP, referred to as ‘increasing negative curvature'. The phases formed by the amphiphilic monoglycerides and fatty acids produced during digestion are therefore strongly driven by the lipid chain length and unsaturation present in the initially consumed formulation lipids. In particular, the presence of *cis-*double bonds in lipid tails has a strong tendency to disrupt lipid packing and drive the structures towards those of greater negative curvature/CPP.

### Measuring the evolution of structure during digestion with synchrotron X-ray scattering

**Figure 2.**
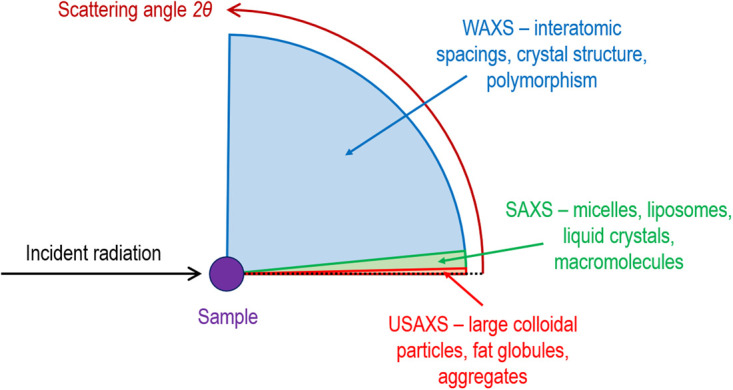
The inverse relationship between scattering angle and the size/types of structural elements probed. WAXS = wide angle X-ray scattering, SAXS = small angle X-ray scattering, USAXS = ultra-small angle X-ray scattering.

Whilst it is possible to identify the various liquid crystalline lipid structures that form during digestion qualitatively using polarised light microscopy [8], unambiguous determination of the structures present is typically achieved using X-ray scattering techniques [6]. X-ray scattering occurs through the interaction of X-rays with domains of varying electron density in a material. The periodic fluctuations in electron density found in a repetitive crystalline structure such as those in the self-assembled lipid phases or the powders of crystalline drugs leads to diffraction, specific angles at which X-rays are scattered strongly through constructive interference of the outgoing waves after interaction with the sample. The angles at which constructive interference occurs are governed by Bragg's Law [9] and lead to the appearance of peaks in scattered X-ray intensity as a function of scattering angle (2*θ*), colloquially referred to as diffraction peaks or ‘Bragg peaks'. The relative peak positions depend on the underlying structure, acting as a characteristic fingerprint for different crystal structures. Examples of the characteristic scattering patterns for four different self-assembled lipid phases are shown in Figure 1 beneath their respective structures [6]. The absolute peak positions are dependent on the wavelength (*λ*) of the X-rays and the interdomain spacing. Smaller molecular length scale structures (∼1–10 Å, such as different polymorphs of crystalline drug powders) lead to wide angle scattering, usually in the form or diffraction patterns from which crystal structures can be determined by indexing the diffraction peaks. Larger colloidal scale structures (∼10–1000 Å, colloidal particles, lipid self-assembly) lead to scattering at smaller angles, either in the form of low angle diffraction patterns from liquid crystals with large interplanar spacings (Figure 1) or more diffuse scattering features close to the unscattered primary X-ray beam. These diffuse small angle scattering features can be modelled to determine low-resolution information on the overall size and shape of colloidal particles [10]. There is often a discrimination in the literature between so-called small-angle X-ray scattering (SAXS) and wide-angle X-ray scattering (WAXS). However, there is no assigned angle that constitutes a definitive crossover between the two and they are essentially the same technique, so in this review the topic is referred to generally as ‘X-ray scattering'. Because the scattering angle varies with wavelength of the incident X-rays, the magnitude of the scattering vector denoted *q* is often used as a wavelength-independent measure of scattering angle (*2θ*), where *q* = (4π/*λ*)sin(*2θ*/2). Figure 2 gives a stylised representation of the types of nano-microscale structuring that can be measured at different scattering angles relative to the incident X-ray beam.

The ability to measure evolving lipid and drug structures on a physiologically relevant timescale has been significantly enhanced by the advent of synchrotron X-ray sources, which present numerous experimental advantages over traditional laboratory sources. Laboratory-based X-ray diffraction instruments are typically limited to a single wavelength (often copper-Kα, *λ* = 1.541 Å), whereas it is usually possible to scan a range of wavelengths precisely at synchrotron sources to move diffraction peaks into a detectable range or to allow the X-rays to penetrate through sample environment walls (e.g. scattering through microfluidic chips) [11]. The extremely high flux of synchrotron sources [12] also confers a major advantage over even the best lab instrumentation. The flux is often 3–4 orders of magnitude greater than a laboratory source, meaning that equivalent information that may take one hour to collect on a laboratory source may be obtained in a fraction of a second [13], even from small quantities of weakly scattering samples. The short acquisition time required to obtain sufficient information to characterise the structure of a material when using a synchrotron source therefore allows for the determination of dynamic changes in systems in response to some change in conditions or under a specific stimulus such as the digestion of lipids in a drug delivery vehicle. A key disadvantage of synchrotron X-rays that should be acknowledged is that there is the strong potential for damage to the sample from high intensity X-ray beams [14]. In the case where a fluidic sample can be subjected to the X-ray beam under continuous flow then damage may be minimised but kinetic studies on static solid samples need appropriate control experiments to ensure that changes in the structure of the sample are due to the stimulus being applied and not radiation damage. The ability to acquire kinetic information on rapidly changing systems using *in situ* sample environments enables deeper understanding of changes to materials on physiological timescales. This is key to the rational development of responsive drug delivery systems, which is the primary focus of this review. The following sections will discuss time-resolved X-ray scattering studies on the digestion of lipid-based drug delivery systems and the observed impacts on lipid and drug structuring.

### Complementary techniques for studying the structural evolution of lipids during digestion

Two important complementary techniques that are also employed in studying the evolving structures of digesting lipids are small angle neutron scattering (SANS) and molecular dynamics (MD) simulations. SANS experiments operate ostensibly in the same manner as SAXS experiments but the sample is exposed to neutron radiation from a nuclear reactor or spallation neutron source (typical *λ* = 1−20 Å). An advantage of using neutron scattering over X-ray scattering is that the contrast between materials leading to the scattering interaction comes from variations in the bound coherent scattering length (*b*) of nuclei in each material [15]. Scattering length density contrast can be enhanced by isotopic substitution of hydrogen (*b* = −3.74 fm) that decreases the coherent scattering power for neutrons, for deuterium (*b* = 6.67 fm) that increases the coherent scattering power for neutrons. This allows particular components of a mixture to be highlighted by tuning the level of deuteration of the aqueous dispersant e.g. deuterated molecules are highlighted in H_2_O and hydrogenous molecules are highlighted in D_2_O [16–18]. In the context of digestion, SANS has been applied most extensively in studying the interaction of simulated intestinal fluids (bile salt/phospholipid mixed micelles and vesicles) with digestive enzymes or simulated digestion product mixtures [19–22]. The main drawback of neutron scattering is the inherently lower radiation flux of neutron sources when compared with synchrotron light sources. This increases the typical acquisition time required for data collection, which thereby reduces the temporal resolution of time-resolved neutron studies relative to synchrotron X-ray measurements. For this reason, many neutron scattering studies are not *in situ* time-resolved measurements of the evolving sample but are *ex situ* steady-state measurements on simulated digesting mixtures [19,22] or those in which the lipase has been inhibited by the addition of an alcoholic inhibitor solution [21]. Solutions to the issue of temporal resolution include improving detector efficiency [23] and the more widespread use of time-of-flight SANS (TOF-SANS) instruments. In TOF-SANS experiments, the scattering of pulses of the whole neutron spectrum coming from the source (as opposed to a continuous monochromatic neutron beam) is measured simultaneously with multiple detector banks encompassing different scattering angles [24]. These instruments therefore provide a wider dynamic *q*-range that can be measured in real time and can also potentially improve the resolution of diffraction peaks at higher *q* values relative to their monochromatic counterparts.

It is increasingly popular for *in vitro* studies on the structure of simulated intestinal fluids during digestion to be complemented by computational modelling and in particular MD simulations [25–27]. Whilst conserving experimental resources, a key limitation of these studies is the complexity of the model system that can be generated for which the MD simulations converge within a reasonable timeframe. To this end, most MD simulations involve variations on coarse-grain modelling [28], dissipative particle dynamics [29] and united atom forcefields [30], rather than all-atom simulations. To maintain the simplicity of the models, where lipid digestion products are introduced in the simulations often only a single representative lipid chain length or digestion product is introduced [20,27,31], rather than the more complex lipid mixtures typically encountered in lipid-based formulations. Nonetheless, MD simulations have been combined with both SAXS [19,32] and SANS [19,20] analyses to support structural models of intestinal fluids during digestion.

## *In situ* studies of lipid digestion relevant to lipid-based drug formulations

**Figure 3.**
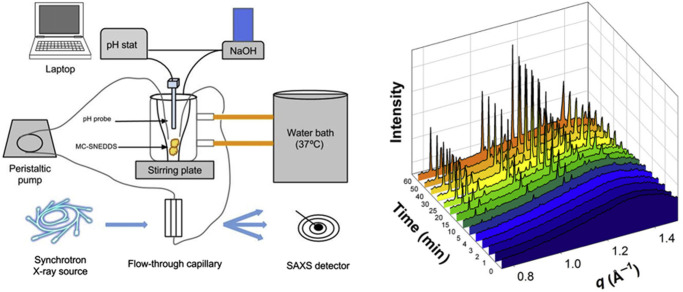
(left) Configuration for *in situ* X-ray scattering measurements during digestion of a self-nano-emulsifying drug delivery system (SNEDDS) and (right) the appearance of diffraction peaks signifying the crystallisation of fenofibrate during digestion of a formulation. Adapted from [60] with permission, future requests for reproduction should be made to Elsevier (https://jpharmsci.org/article/S0022-3549(15)00066-0/fulltext).

Whilst it has been known for decades that monoglycerides and fatty acids self-assemble with rich lipid polymorphism into an array of systematically varying structures [33,34], the observation of these processes during the lipolysis of triglycerides has only recently been reported. The major studies focussing on studying self-assembly during lipid digestion using time-resolved X-ray scattering approaches are highlighted in Table 1. Earlier promising studies using steady-state measurements on laboratory-based instruments [35–38] inspired the development of synchrotron-based lipolysis models that enable high resolution diffraction studies during lipolysis of a diverse range of dilute lipid-based systems including pharmaceutical lipid formulations such as medium chain triglyceride (MCT) [39,40], milk [41–43], infant formula [44], mayonnaise [45] and fish oil supplements [46]. These studies are typically conducted by coupling a pH-stat model of lipid digestion to the synchrotron X-ray scattering measurement (Figure 3). In the pH-stat experiment, the pH of the digesting medium is set to a fixed value (often pH 6.5–7.0 to be representative of the small intestine) and free fatty acids released during lipolysis are titrated using a base solution to maintain a constant pH. This allows the kinetics and extent of digestion to be monitored whilst simultaneously a portion of the sample is circulated through the synchrotron X-ray beam, allowing X-ray scattering patterns to be measured in real time.

**Table 1:** Time-resolved X-ray scattering studies of lipid systems of pharmaceutical and food relevance during digestion

Enzyme	Substrate	Change in lipid structure	Refs
Pancreatin	Triolein	Forms micellar cubic phase (I_2_) during digestion	[38]
Pancreatin	MCT	Vesicles (L_α_) formed at a critical extent of digestion	[38,39]
Pancreatin	Bovine milk	Initially lamellar calcium soaps, followed by inverse micellar cubic (I_2_), hexagonal (H_2_) and bicontinuous cubic (V_2_) phases in sequence	[41,43]
Pancreatin	Human milk	Initially lamellar calcium soaps followed by micellar cubic (I_2_) phase at completion	[42]
Pancreatin	Infant formula	Depends on the brand/lipid composition, some recapitulate the micellar cubic (I_2_) phase of human milk, some form hexagonal (H_2_) phases and others just form calcium soaps	[44]
Pancreatin	Mayonnaise	Similar to bovine milk, inverse micellar cubic (I_2_), inverse hexagonal (H_2_), bicontinuous cubic (V_2_) phases all seen in succession	[45]
Pancreatin	Krill oil and astaxanthin	Inverse hexagonal (H_2_) and lamellar phases	[46]
Pancreatin	Milk-mimicking triglyceride mixtures	Same as bovine and human milk with different mixtures of homo-triglycerides and commercial oils	[7,47]
Pancreatin	Cyclopropanated monoglycerides	Most lipids transitioned to structures with greater negative curvature (from V_2_/H_2_ towards H_2_/I_2_) during digestion	[49]
Pancreatin	Glyceryl dioleate + soy PC	Transition from inverse micellar (I_2_) phase to lamellar (L_α_) via inverse hexagonal (H_2_) and inverse bicontinuous cubic (V_2_) phases	[50]
Pancreatin	Phytantriol + tributyrin particles	Transition from disordered inverse micellar phase to inverse bicontinuous cubic (V_2_) phase upon digestion of tributyrin	[11]
Pancreatin and phospholipase A2	Glyceryl dioleate + soy PC	Inverse micellar cubic (I_2_) phase to either disordered inverse micellar phase (PLA2) or lamellar phase (pancreatin)	[51]
Phospholipase C	Soy PC and egg PE	Headgroup cleaved, causes lamellar (L_α_) to inverse hexagonal (H_2_) transition for egg PE only	[52]
Fungal lipase	Monoolein cubosomes	Inverse bicontinuous cubic (V_2_) to inverse hexagonal (H_2_) and vesicular (L_α_) phases	[53]
Fungal lipase	Monoolein/sodium oleate vesicles	Spherical vesicles/tubular (L_α_) structures become irregular in shape with deformed lipid bilayers	[53]
Fungal lipase	Phytantriol cubosomes	Digestion of stabilisers triggers inverse bicontinuous cubic (V_2_) phase to inverse hexagonal (H_2_) phase transition	[54]
Bacterial Lipase	Monoolein cubosomes	Inverse bicontinuous cubic (V_2_) phase to lamellar phase	[55]
Invertase	Vesicles containing sucrose laurate	Vesicles (L_α_) to bicontinuous cubic (V_2_) phase	[56]

In general, the self-assembly of lipids induced by digestion processes can be rationalised by the critical packing parameter concept by considering the lipid composition in terms of lipid chain length/unsaturation and factoring in for changes in effective headgroup size during digestion. Lipid self-assembly generally progresses from structures of higher interfacial curvature (inverse micellar phases) towards those of lower interfacial curvature (bilayer type phases) or *vice versa* as the CPP of the lipid digestion products evolves. In the context of typical lipid-based drug delivery systems, triglyceride mixtures richer in saturated acyl chains tend to form structures of lower interfacial curvature during digestion [38,41,43–45], whilst triglyceride mixtures richer in unsaturated acyl chains form structures of higher interfacial curvature [38,39,42,44]. The presence of calcium ions in the digesting medium also has a strong tendency to influence the lipid structuring by forming insoluble calcium soaps as fatty acids are released during digestion. These calcium soaps have lamellar structures [43], with the characteristic spacing between the sheets being indicative of the predominant acyl chain length in the soap.

Understanding how triglyceride composition directly affects subsequent lipid structuring provides opportunities to reverse engineer self-assembly behaviour in the gastrointestinal tract through the selection of lipid compositions that will target certain structures of either high or low interfacial curvature during digestion [7,47]. This could be used to trigger drug release, but there are also likely to be consequences for particle interactions with other gut components and structures such as bile, mucus and the gut microbiome. Linking the structural changes during digestion to lipophile delivery is somewhat in its infancy, but coupling such studies to e.g. cell systems [48] to enable lipid absorption is the next stage of development towards more biorelevant lipolysis and structural studies.

## Drug solubilisation and crystallisation during digestion of lipid systems

The process of lipid digestion can be useful in the context of drug delivery, where digestion products can act to dissolve and carry lipophilic compounds through the gastrointestinal tract and enable the absorption of what would otherwise be intractable insoluble substances [57]. Most pharmaceutical scientists are familiar with the appearance of X-ray diffraction patterns from pharmaceutical powders. The time required for acquisition of a powder pattern on a lab-based X-ray instrument is typically 15–20 min. The high flux available at synchrotron sources, together with large area detectors means that scattering from very low concentrations of crystalline material dispersed in a formulation can be determined in fractions of a second. This enables measurement of dissolution/amorphisation, precipitation and polymorphic transformation of drugs in dilute suspension on timescales relevant to digestion processes [58,59]. *In vitro* digestion models can be coupled to X-ray scattering to study the solid state structure of drugs *in situ* in digesting lipid systems (illustrated schematically on the left side of Figure 3). Detection of drug crystals at concentrations <0.5 mg/ml has also meant that pharmaceutically relevant ratios of drug to lipid/formulation can be studied. During digestion of a lipid formulation, there are three main outcomes that may eventuate with respect to the fate of the drug that are described in the following sections.

### The drug is more soluble in the undigested formulation lipids than in the digestion products

Drug will precipitate during digestion and characteristic diffraction peaks will grow over time at the corresponding angles where the peaks occur in a powder X-ray diffraction (XRD) measurement. An example of this behaviour where drug precipitation from a digesting SMEDDS formulation containing fenofibrate is shown in the right side of Figure 3 [60]. The timescale of precipitation is a matter of minutes, which makes it difficult to obtain kinetic information from more traditional XRD or analytical separation approaches. This type of behaviour may indicate poor performance of the formulation *in vivo* as the presence of the precipitated crystalline drug is usually correlated with reduced oral bioavailability [61].

### The drug undergoes a polymorphic transformation triggered by lipid digestion

The transformation of solid drug substances to different polymorphic forms upon exposure to different aqueous environments is well documented. The transformation is often mediated by a higher solubility of one polymorphic form of the drug in the surrounding digesting media, leading to dissolution and recrystallisation of the drug in the more stable, less soluble, polymorphic form. While the phenomenon is well studied in static biorelevant media during dissolution studies [62] there are few reports of such behaviour during *in situ* digestion (one example in the literature involves tolfenamic acid, however it should be noted that this was formulated in an indigestible lipid-based formulation [63]) and almost none have been measured using synchrotron X-ray scattering during lipid digestion.

**Figure 4.**
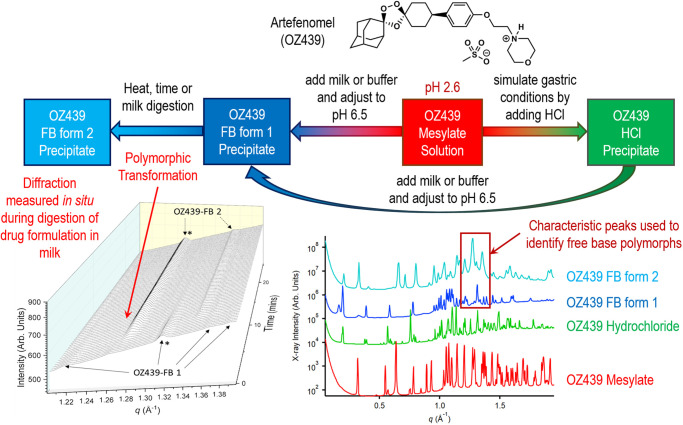
Complex salt and polymorphic transformations of artefenomel (OZ439 mesylate) on exposure to biorelevant media and during digestion in milk. The bottom right panel illustrates the different diffraction patterns for the mesylate salt, the poorly soluble hydrochloride salt and the free base forms (FB form 1 and FB form 2) of artefenomel. The bottom left panel illustrates the slow transformation over 10 min from the free base form 1 (FB 1) to free base form 2 (FB 2) polymorph in digesting milk measured using *in situ* synchrotron X-ray scattering. Adapted from [67] with permission, future requests for reproduction should be made to ACS publications (https://pubs.acs.org/doi/10.1021/acs.molpharmaceut.8b00541).

Recent studies have however shown the power of such measurements in understanding complex behaviour of drugs during the digestion of lipid-based formulations. The antimalarial drug artefenomel (OZ439) is a drug belonging to the ozonide family (Figure 4) [64], which when paired with ferroquine (Figure 6), shows potential as a single dose cure for malaria [65]. Artefenomel is amphiphilic, forming micelles and other aggregate structures in aqueous solution [66] and was developed as a mesylate salt to optimise its apparent aqueous solubility. However, this exposed the risk of precipitation upon introduction to gastrointestinal environments where formation of a poorly soluble hydrochloride salt or free base form results in drug precipitation. Using time-resolved X-ray scattering, this behaviour has been demonstrated during digestion of milk and infant formula as potential lipid-based formulations for use in low-income settings [67]. Formation of the hydrochloride salt from the mesylate salt exposed to hydrochloric acid solution is extremely rapid, and neutralisation to intestinal pH induces a further transformation to the poorly soluble free base form 1 (FB 1). There is then a transformation of FB 1 to a second free base form (FB 2) that occurs during digestion, concurrent with solubilisation as described in the next section.

### The drug is more soluble in the digestion products than the undigested formulation lipids

There is a paradigm in the lipid-based formulation field that drug must be in solution in the undigested formulation and that much effort needs to be spent on developing the formulation to maintain drug in solution for as long as possible to prevent drug precipitation [68]. From a pharmaceutical development perspective this is a high-risk strategy akin to the development of amorphous drug formulations, where interpatient variability and storage conditions can lead to highly variable precipitation behaviour with consequent impacts on bioavailability. The digestion of lipids has evolved not only as a means for our body to absorb lipids as energy and structural components from a nutrition perspective but also as a means of enabling absorption of lipophilic nutrients in our diet. Thus, perhaps there is an alternative paradigm of not trying to fight digestion to maintain a thermodynamically unfavourable state, but to understand and use the digestion of lipids as a tool to enable absorption of otherwise insoluble drug substances. It has been shown that administration of drug and lipid separately [69,70] is as effective in many cases as formulation of the drug in the lipid as a lipid-based formulation. The co-administration of drug with lipid presents opportunities especially for drugs that have poor solubility in the undigested lipids, or poor stability in the lipid formulation. The concept also allows for the separate supply of solid dose forms and lipid formulations, which may be lipids in a capsule, or could be an otherwise available liquid vehicle such as milk or infant formula.

**Figure 5.**
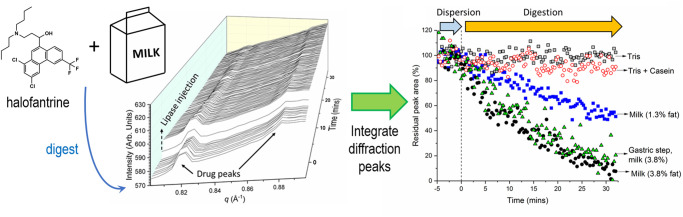
Digestion of full cream milk containing halofantrine results in complete solubilisation of the drug, whereas lower fat content milk or casein solutions do not. Dispersion is the period during which the drug and milk are mixed prior to addition of lipase to initiate lipid digestion. Adapted from [57] with permission, future requests for reproduction should be made to Elsevier (https://www.sciencedirect.com/science/article/pii/S0168365918306060?via%3Dihub).

For weakly basic drugs the formation of fatty acids during digestion can be crucial in driving the solubilisation of drug through formation of lipophilic ion pairs [57]. The solubilisation of solid crystalline drug during digestion has been shown for pharmaceutical lipids [71], milk [57,67,72] and infant formula [73]. Halofantrine as a model drug was shown to be fully solubilised into full cream milk during digestion but not lower fat milk or casein solutions (Figure 5) [57].

**Figure 6.**
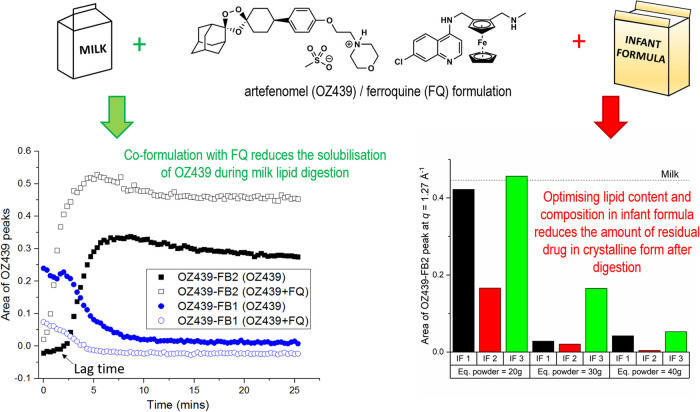
Solubilisation of artefenomel (OZ439) during digestion of OZ439 mesylate/ferroquine formulations in milk (left) and residual crystalline OZ439 after digestion of the same drug combination in three different infant formulas (right). The left panel also illustrates the transformation between the two free base forms of artefenomel during digestion. The dependence of solubilisation on fat content in the different infant formulas is also illustrated on the right, note that lower bar height indicates greater drug solubilisation. Adapted from [72,73] with permission, future requests for reproduction should be made to ACS publications (https://pubs.acs.org/doi/10.1021/acs.molpharmaceut.8b01333 & https://pubs.acs.org/doi/abs/10.1021/acs.molpharmaceut.0c00475).

In the case of artefenomel introduced in the previous section, the digestion of milk lipids led to the rapid solubilisation of artefenomel FB 1, which subsequently reprecipitated as the FB 2 polymorph (Figure 6, left panel). Full digestion of the milk lipids did not enable complete solubilisation of artefenomel FB 2 and co-formulation with ferroquine reduced the solubilisation of FB form 2 [72]. Importantly, subsequent studies showed that the selection of an infant formula with an optimal lipid composition and lipid:drug ratio enabled full solubilisation of artefenomel during digestion (Figure 6, right panel) [73].

## Conclusion

Lipids and lipid-based formulations undergo complex changes in chemical and physical structure during digestion. By selecting appropriate formulation lipids, drug delivery systems can be designed to control nanostructure and drug release using digestion as a trigger. To enable the rational design of optimal lipid-based drug delivery systems the physical-chemical complexity in these systems should be harnessed, which in turn requires a clear understanding of the structural properties of lipids during these transformations. X-ray scattering is a key technique in this regard but it is only through the use of a synchrotron source that we are able to elucidate changes in dilute dispersed systems *in situ* on physiologically meaningful timescales. The use of a synchrotron source also allows access to the solid-state characteristics and transformations of drugs incorporated into lipid-based formulations which can provide crucial quantitative information to lead formulation design and maximise drug solubilisation during digestion. The increased awareness of these techniques and efforts at developing methodologies for *in situ* probing of lipid self-assembly and consequent impacts on drug solubilisation or release will facilitate development of new more complex formulation approaches from a rational standpoint.

## Perspectives

- Lipid-based formulations for oral delivery of poorly water-soluble drugs often contain digestible lipids but the impacts of digestion on lipid structuring and drug solubility are often neglected in the assessment of their efficacy. Recent developments in synchrotron X-ray scattering techniques have enabled time-resolved studies on lipid and drug structuring *in situ* during lipid digestion in real time, providing a framework for the rational design of drug delivery systems triggered by lipid digestion.
- When designing lipid-based formulations for drugs, it is often assumed that maximising and maintaining the solubility of the drug in the undigested formulation is the best pathway to improving bioavailability. However, by optimising the composition of the formulation lipids it is possible to exploit the natural process of lipid digestion to control lipid self-assembly and drug solubilisation in the gastrointestinal tract and thereby promote drug absorption.
- Future studies will begin to examine the interactions of digesting lipid-based formulations with increasingly complex biorelevant environments. Real-time structural investigations into the interactions between digesting lipid-based formulations and mucus, cells and the gut microbiome remain in their infancy.

## Open Access Statement

Open access for this article was enabled by the participation of Monash University in an all-inclusive Read & Publish pilot with Portland Press and the Biochemical Society under a transformative agreement with CAUL.

## Abbreviations

CPP: critical packing parameter
MCT: medium chain triglyceride
MD: molecular dynamics
SANS: small angle neutron scattering
SAXS: small-angle X-ray scattering
TOF-SANS: time-of-flight SANS
WAXS: wide-angle X-ray scattering
XRD: X-ray diffraction
